# Influence of Glycosylation Inhibition on the Binding of KIR3DL1 to HLA-B*57:01

**DOI:** 10.1371/journal.pone.0145324

**Published:** 2015-12-17

**Authors:** Wilhelm Salzberger, Wilfredo F. Garcia-Beltran, Haley Dugan, Supreetha Gubbala, Camille Simoneau, Simon B. Gressens, Stephanie Jost, Marcus Altfeld

**Affiliations:** 1 Department of Virus Immunology, Heinrich-Pette-Institut, Leibniz Institute for Experimental Virology, Hamburg, Germany; 2 Ragon Institute of MGH, MIT, and Harvard, Cambridge, Massachusetts, United States of America; University of Sydney, AUSTRALIA

## Abstract

Viral infections can affect the glycosylation pattern of glycoproteins involved in antiviral immunity. Given the importance of protein glycosylation for immune function, we investigated the effect that modulation of the highly conserved HLA class I *N*-glycan has on KIR:HLA interactions and NK cell function. We focused on HLA-B*57:01 and its interaction with KIR3DL1, which has been shown to play a critical role in determining the progression of a number of human diseases, including human immunodeficiency virus-1 infection. 721.221 cells stably expressing HLA-B*57:01 were treated with a panel of glycosylation enzyme inhibitors, and HLA class I expression and KIR3DL1 binding was quantified. In addition, the functional outcomes of HLA-B*57:01 *N*-glycan disruption/modulation on KIR3DL1ζ^+^ Jurkat reporter cells and primary human KIR3DL1^+^ NK cells was assessed. Different glycosylation enzyme inhibitors had varying effects on HLA-B*57:01 expression and KIR3DL1-Fc binding. The most remarkable effect was that of tunicamycin, an inhibitor of the first step of *N*-glycosylation, which resulted in significantly reduced KIR3DL1-Fc binding despite sustained expression of HLA-B*57:01 on 721.221 cells. This effect was paralleled by decreased activation of KIR3DL1ζ^+^ Jurkat reporter cells, as well as increased degranulation of primary human KIR3DL1^+^ NK cell clones when encountering HLA-B*57:01-expressing 721.221 cells that were pre-treated with tunicamycin. Overall, these results demonstrate that *N*-glycosylation of HLA class I is important for KIR:HLA binding and has an impact on NK cell function.

## Introduction

Natural killer (NK) cells are part of the innate immune system, and serve as a first line of defense against intracellular pathogens and malignantly transformed cells. NK cells can lyse target cells via directed secretion of perforin and granzyme, antibody-dependent-cell-mediated-cytotoxicity and Fas/FasL-mediated cytotoxicity [1], but can also activate and recruit other immune cells via secretion of pro-inflammatory cytokines and chemokines. The importance of NK cell function is highlighted in the setting of impaired NK cell function or NK cell deficiencies, which are associated with increased susceptibility to infections with viruses and intracellular bacteria [1–4]. NK cell activity is regulated via a number of activating and inhibitory receptors, including killer cell immunoglobulin-like receptors (KIRs) [1, 5], which recognize human leukocyte antigen (HLA) proteins. Binding of KIRs to HLA class I molecules is determined by the respective HLA class I and KIR allotypes, as well as the sequence of the HLA class I-presented peptides [6, 7]. While binding of inhibitory KIRs to HLA class I molecules results in NK cell inhibition, engagement of activating KIRs or loss of inhibitory KIR binding can lead to NK cell activation [8, 9]. The KIR-HLA interaction has been demonstrated to have an important impact on viral infections, including human immunodeficiency virus-1 (HIV-1). In HIV-1 positive individuals, KIR3DL1^+^ NK cells expanded preferentially in the presence of HLA-Bw4-80I, the ligand class for KIR3DL1 [10, 11].

Protein glycosylation is a post-translational modification occurring in the endoplasmatic reticulum (ER) and Golgi apparatus. In multiple enzymatic steps, a complex oligosaccharide (*i*.*e*. glycan) is synthesized in the ER, transferred to a specific receptor sequence on its target protein, and subsequently cropped and remodeled in the ER and Golgi [12]. In the case of asparagine *N*-glycosylation, the target amino acid sequence on the respective proteins is N-X-S/T, where the glycan is bound to the asparagine. It is estimated that over 7,000 distinct glycan structures can be generated in mammals [13], suggesting a wide range of functional properties, which can be modulated by differential glycosylation. On HLA-class I, the glycan at position *N*86, which is located close to the Bw4 motive critical for binding of KIR3DL1 to its ligands, is highly conserved and present on practically all HLA-class I allotypes [14, 15]. Here we use a panel of glycosylation inhibitors to examine the effects of glycosylation modifications on KIR3DL1:HLA-B*57:01 interactions and their potential effect on NK cell function, with a more in-depth characterization of the effect tunicamycin (TUN)—an antibiotic that can completely block *N*-glycosylation in eukaryotic cells by preventing the linkage of the glycan to the asparagine [16, 17]—and castanospermine (CSP)—a α-glucosidase I and II inhibitor able to inhibit the glycan trimming in the ER necessary for the assembly of a fully functional glycan [17, 18].

## Materials and Methods

### Target Cells

HLA class I-deficient 721.221 cells (referred to as ‘221 cells’ or ‘221s’ hereafter) stably expressing HLA-B*08:01 or HLA-B*57:01 were provided by Christian Brander (Ragon Institute of MGH, MIT and Harvard, Cambridge, MA) as previously described [19]. All cells were cultured at 37°C with 5% CO_2_ in RPMI 1640 medium (Sigma-Aldrich) supplemented with 10% heat-inactivated fetal bovine serum (FBS) (Biochrom AG), 2500 U/mL penicillin and 2500 μg/mL streptomycin (Sigma-Aldrich) [20] unless stated otherwise. This media was referred to as ‘R10’.

### Glycosylation inhibition screening and titration

Glycosylation inhibition was performed by incubating target cells for 24 h at 37°C/5%CO_2_ in R10 supplemented with different glycosylation inhibitors: 5 mM of 1-deoxynojirimycin (DNM) (Tocris Bioscience), 500 μM of australine (AUS) (Santa Cruz Biotechnology), 2 mM of castanospermine (CSP) (Tocris Bioscience), 100 μM of kifunensine (KIF) (Tocris Bioscience), 500 μM of 3F_ax_-peracetyl-Neu5Ac (STI) (Millipore), 100 μM of swainsonine (SWA) (Tocris Bioscience), 0.5μg/mL of tunicamycin (TUN) [21] (Sigma-Aldrich Chemie GmbH) or phosphate buffered saline (PBS) as a control. Optimal concentrations of glycosylation inhibitors were determined by using effective concentrations reported in the literature as well as lower and higher doses. Readouts measured were cell death of target cells and CD69 expression of KIR3DL1ζ-Jurkat cells after coincubating with glycosylation inhibitor treated HLA-B*57:01-expressing target cells (see Jurkat reporter cell assay methods).

### Quantification of HLA class I expression and KIR3DL1 binding

For quantification of HLA class I cell surface expression, target 221 cells (1.5 × 10^5^ per well) were treated with TUN, CSP, or PBS for 24 h. After washing, cells were stained with either biotinylated anti-HLA-Bw4 antibody (One Lambda) followed by secondary staining with streptavidin-BV421 (BioLegend), or anti-pan-HLA class I-PE antibody (clone: W6/32) (BioLegend). For KIR-Fc binding assessment, target cells (1.5 × 10^5^ per well) were grown for 48 h in modified media prior to KIR-Fc staining to reduce background staining. Modified media consisted of custom-made Advanced RPMI-1640 (Life Technologies) deficient in magnesium sulfate, zinc sulfate, and copper (II) sulfate supplemented with 10% dialyzed FBS (Gibco), 2 mM L-glutamine (Gibco), 100 U/mL penicillin (Gibco), 100 U/mL streptomycin (Gibco), 407 μM magnesium chloride (Sigma-Aldrich), 3.03 μM zinc chloride (Sigma-Aldrich), and 5 nM copper (II) chloride (Sigma-Aldrich), which was mixed with 150 mM sodium chlorate (Sigma-Aldrich) at a 5:1 volume ratio. During the last 24 h, TUN, CSP or PBS was added to the medium. After washing, cells were stained for 45 min with 25 μg/mL KIR3DL1-Fc (R&D) at 4°C while shaking, followed by a secondary staining with anti-hlgG(Fc)-PE (Life Technologies) for 30 min at 4°C while shaking. After staining, cells were fixed using 4% paraformaldehyde/PBS (Affymetrix) and flow cytometry analysis was performed on an LSR-II (BD).

### Jurkat reporter cell assay

Jurkat cells (clone E6.1; ATCC) were cultivated in R10 medium. 24 h before performing experiments, 10% fetal bovine serum was added to the culture to reduce background activation. Jurkat cells stably expressing KIR3DL1ζ, a chimeric receptor composed of the extracellular and transmembrane domains of KIR3DL1 linked to the cytoplasmic tail of CD3ζ, which we denote as ‘KIR3DL1ζ‘, were produced via lentiviral transduction/transfection. Briefly, gene constructs were designed and ordered via GeneArt (Life Technologies), and cloned into a lentiviral transfer vector containing an SFFV promotor and IRES-driven puromycin resistance. HEK293T cells (ATCC) were transfected with a VSV-G envelope vector (pHEF-VSVG, NIH AIDS Reagent Program), HIV-1 gag-pol packaging vector (psPAX2, NIH AIDS Reagent Program), and the transfer vector containing KIR3DL1ζ. Lentivirus-containing supernatants were harvested 72 h after transfection and used to transduce Jurkat cells, which were subsequently selected in R10 containing 1 μg/mL puromycin and sorted for gene expression by flow cytometry. In these KIR3DL1ζ^+^ Jurkat cells, ligand engagement by KIR3DL1ζ results in an activating signal that triggers CD69 expression, making them a suitable reporter cell system. Prior to coincubation with target cells, Jurkat cells were cultured for 24 h in R10 medium supplemented with an additional 10% fetal bovine serum. 221 cells expressing HLA-B*08:01, HLA-B*57:01, or no HLA class I underwent glycosylation inhibition and were washed twice. Target cells were washed to remove glycosylation inhibitors and then co-incubated with either KIR3DL1ζ^+^ or untransduced Jurkat cells at a reporter-to-target cell ratio of 1:1 (1 × 10^5^ each) for 2.5 h at 37°C/5%CO_2_. Cells were then washed, stained with anti-CD3-PerCP-Cy5.5, anti-CD69-BV421, and anti-KIR3DL1-APC (all from BD) for 30 min at 4°C, fixed with 4% paraformaldehyde/PBS, and analyzed on an LSR-Fortessa (BD). KIR3DL1ζ-transduced Jurkat cells were gated for KIR3DL1 expression, classifying them either as KIR3DL1ζ^low^, KIR3DL1ζ^dim^ or KIR3DL1ζ^bright^.

### NK cell cloning and degranulation assay

Primary human NK cells isolated from peripheral blood mononuclear cells (PBMCs) of healthy donors were cloned by limiting dilution in the presence of feeders and maintained in NK cell cloning medium consisting of R10 supplemented with 5% human serum (Sigma-Aldrich), 1X MEM-NEAA (Gibco), 1X sodium pyruvate (Gibco), 100ug/mL Kanamycin, 200 U/mL IL-2 (AIDS Reagent Program, NIH) using a protocol adapted from a previously reported method [22]. Briefly, NK cells were isolated from peripheral blood mononuclear cells (PBMCs) from a *KIR3DL1* ^+^ *HLA-Bw4* ^+^ donor via magnetic negative selection (NK cell isolation kit from Miltenyi), added to a mix of irradiated feeders consisting of freshly isolated allogeneic (PBMCs) combined with log-phase-growth RPMI 8866 cells (Sigma-Aldrich) at a 10:1 ratio in cloning medium containing 1 μg/mL phytohaemagglutinin (PHA; Fisher) and mixed thoroughly before plating at 100 μL/well (0.5 NK cell/well) in 96-well plates and incubated for 14 days at 37°C/5% CO_2_. After 14 days, wells that had outgrowth of cells were transferred to 48-well plates and maintained in NK cell cloning medium with frequent media exchange (approximately every 3 days). Cells were phenotyped by flow cytometry to assess NK cell marker (e.g. CD56 and CD16) and KIR3DL1 expression. Degranulation assays were performed by co-incubating 5 × 10^4^ NK cells with 2.5 × 10^5^ target cells (effector-to-target cell ratio of 1:5) in 200 μL of R10 containing 3 μL of anti-CD107a-PE-Cy7 antibody (BioLegend) for 2 h at 37°C/5% CO_2_, and subsequently staining with anti-CD56-BV605 and anti-CD16-BV785 antibodies (both from BioLegend) for 30 min at 4°C, and then fixed with 4% paraformaldehyde/PBS. Flow cytometric analysis was performed on a BD LSRFortessa.

### Ethics

PBMCs from healthy donors were obtained and frozen after obtaining written informed consent following procedures approved by the Partners Human Research Committee (ethics committee) and the Institutional Review Board of Massachusetts General Hospital. HLA-I types of selected human samples were determined prior to this study by high-resolution HLA-I typing performed at the HLA-typing laboratory of the National Cancer Institute, National Institutes of Health. KIR types were determined prior to this study by Sanger sequencing in the laboratory of Mary Carrington.

### Statistics

Flow data was analyzed in FlowJo v10 (FlowJo LLC, Ashland, Oregon). Statistical analyses were performed using GraphPad Prism 6.0c (GraphPad Software Inc., La Jolla, California, USA). Normality tests (Kolmogorov-Smirnov test with Dallal-Wilkinson-Lilliefor P value) were performed on all data sets, statistical significance was calculated via one-way ANOVA, and multiple comparisons were corrected using Tukeys’s test if not stated otherwise. All graphs show mean ± SD if not stated otherwise in the figure legends. Significance levels were defined as *<0.05, **<0.01, ***<0.001 and ****<0.0001.

## Results

### Effects of glycosylation enzyme inhibitors on HLA expression and KIR-Fc binding

**Fig 1:**
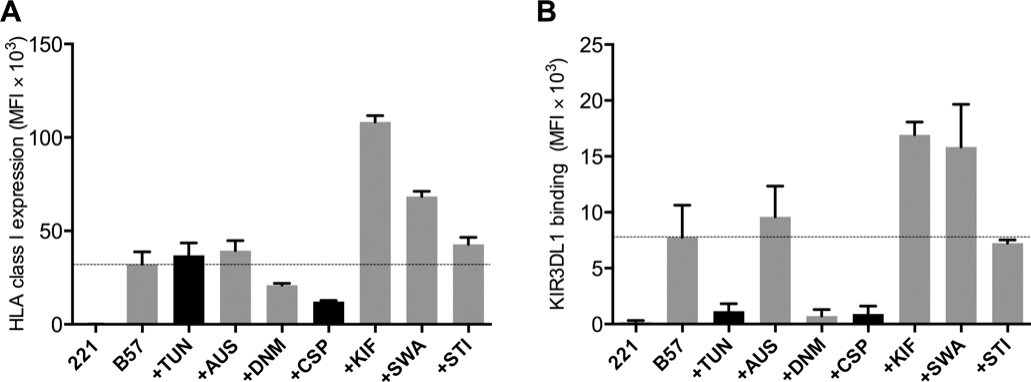
Glycosylation inhibitor screening and titration: (A) Median fluorescence intensity (MFI) of Bw4 staining of untransfected 221 cells (221) and HLA-B*57:01 transfected 221 cells (B57) treated with a panel of glycosylation inhibitors (n = 2) (B) MFI of KIR-Fc staining of untransfected 221 cells (221) and HLA-B*57:01 transfected 221 cells (B57) treated with a panel of glycosylation inhibitors (n = 2)

In order to examine the effect of different commercially available glycosylation inhibitors on KIR:HLA interactions, we examined their effect on HLA-B*57:01 expression on HLA-B*57:01-transduced 221 cells and on KIR3DL1-Fc binding to those cells. Inhibitors that targeted Golgi-resident α-mannosidase enzymes (KIF, SWA) increased HLA-B*57:01 expression, which resulted in minimally increased (MFI of 32220 vs. MFI of 36900; *p* = 0.9456) KIR3DL1 binding (Fig 1A and 1B). The Golgi-resident sialyl transferase inhibitor (STI) tested, however, did not result in any significant change in HLA-B*57:01 expression or KIR3DL1 binding. Inhibitors that targeted ER-resident α-glucosidase II (CSP, DNM), which is an enzyme necessary for the last removal of glucose from *N*-glycans to allow anterograde transport to the Golgi, dramatically reduced HLA-B*57:01 expression and abrogated KIR3DL1 binding. The ER-resident α-glucosidase I-specific inhibitor (AUS) minimally increased HLA-B*57:01 expression or KIR3DL1 binding (Fig 1A). However, in the case of TUN, we observed a major decrease in KIR3DL1-Fc binding while HLA-B*57:01 surface expression was increased slightly (Fig 1B). Thus, after preliminary testing of the effects that several different glycosylation inhibitors had on HLA class I expression and KIR binding, we decided to focus on TUN, the only inhibitor tested that did not decrease HLA class I surface expression but dramatically reduced KIR3DL1-Fc binding, and that had the unique function of completely inhibiting that addition of *N*-glycans.

We subsequently performed a titration assay in order to identify the minimal amount of glycosylation inhibitors required to observe a strong functional effect on the binding of KIR3DL1ζ^+^ Jurkat cells to HLA-B*57:01-expressing target 721.221 cells while keeping target cell death to a minimum. We observed a dose-dependent effect of both TUN and CSP on KIR3DL1ζ Jurkat cell signaling, with the strongest measured effect (*i*.*e*. decreased activation of KIR3DL1ζ^+^ Jurkat cells as measured by CD69 expression) corresponding to the highest treatment dose used in previous studies: 0.5 μg/mL of TUN (S2A Fig) or 2 mM of CSP (S2B Fig). Higher doses of glycosylation inhibitors increased cell toxicity to a degree that interfered with our assay, as cell death of target cells rose dramatically if the concentrations of glycosylation inhibitors were increased over the recommended concentration. Lower doses of inhibitors increased signaling of KIR3DL1ζ Jurkat cells after coincubation, indicating a loss of inhibition of target cell glycosylation. Thus, we concentrated on the glycosylation inhibitor TUN at a concentration of 0.5 μg/mL, representing a similar concentration as used in previous studies on the effects of TUN on HLA class I glycosylation [23, 24].

### HLA class I *N*-glycan is necessary for KIR3DL1 binding to HLA-B*57:01

We next sought to quantitatively assess the effects of TUN on HLA-B*57:01 surface expression and KIR3DL1 binding. Wildtype 221 cells and 221 cells expressing HLA-B*57:01 were incubated overnight with PBS, CSP or TUN, stained with anti-Bw4, anti-pan-HLA class I antibodies and KIR3DL1-Fc and assessed via flow cytometry.

**Fig 2:**
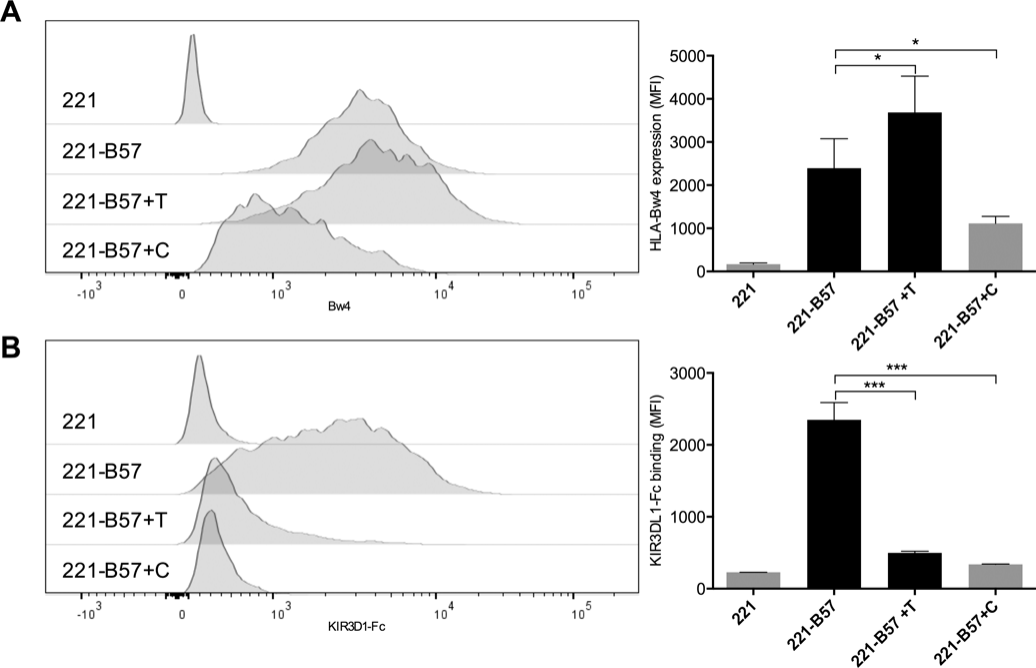
*N*-glycosylation inhibition increases HLA-B*57:01 surface expression while abrogating KIR3DL1-Fc binding: Anti-HLA-Bw4 antibody staining (A) and KIR3DL1-Fc staining (B) was performed on 221-HLA-B*57:01 cells (221-B57) treated with TUN (+T), CSP (+C), or PBS and untransduced 221 cells (221). Representative histograms are on left-sided panels and data representing five technical replicates are presented as bar graphs on right-sided panels.

Co-incubation of 221-HLA-B*57:01 cells with TUN (+T) for 24 h significantly increased HLA-B*57:01 expression as measured by anti-HLA-Bw4 antibody staining (1.5-fold HLA-Bw4 MFI increase, *p* = 0.02; 2.0-fold W6/32 MFI increase, p < 0.0001) (Fig 2A, S1 Fig). In contrast, co-incubation with CSP (+C) significantly decreased HLA expression (2.1-fold HLA-Bw4 MFI decrease, p = 0.02; 1.7-fold W6/32 MFI decrease, p < 0.0001). Since both the staining with the anti-HLA-Bw4 antibody as well as the staining with the anti-pan-HLA class I antibody, which only recognizes HLA class I proteins that are properly folded and bound to β_2_-microglobulin, were similarly affected my the inhibition of glycosylation, we believe that the HLA-B*57:01 proteins expressed at the cell surface were indeed properly folded HLA-B*57:01 complexes and not free heavy chains.

Sustained expression of HLA-B*57:01 on 221-B*57:01 cells following TUN treatment enabled assessment of the consequences of HLA class I de-glycosylation on KIR3DL1 binding to HLA-B*57:01. Despite enhancement of overall HLA-B*57:01 expression, TUN abrogated KIR3DL1-Fc binding. Cells treated with TUN bound KIR3DL1-Fc significantly less than untreated cells (4.7-fold KIR-Fc MFI decrease, *p* < 0.0001). 221-HLA-B*57:01 cells treated with CSP also exhibited reduced KIR3DL1-Fc binding (6.9-fold KIR-Fc MFI decrease, *p* < 0.0001) (Fig 2B), which was expected given the reduced surface expression of HLA-B*57:01 on CSP-treated cells. Taken together, these data demonstrate that the presence of *N*-glycosylation is critical for KIR3DL1 binding to HLA-B*57:01.

### HLA class I *N*-glycan is necessary for functional signaling through KIR3DL1 in KIR3DL1ζ^+^ Jurkat cells

**Fig 3:**
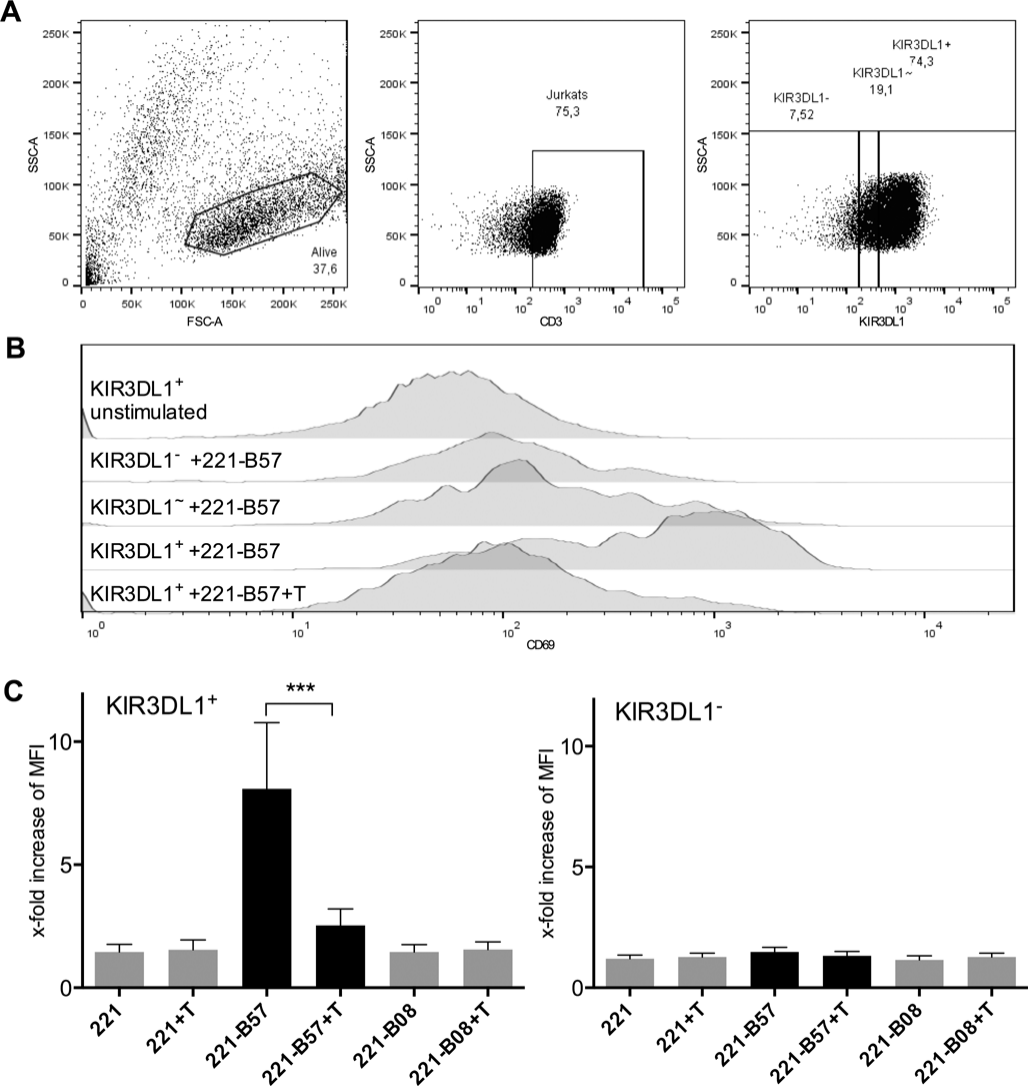
TUN treatment HLA-B*57:01 221 cells abrogates binding to KIR3DL1ζ-Jurkat cells: (A) Gating of Jurkat cells by size (SSC = side scatter; FSC = forward scatter), CD3 expression and KIR3DL1 expression (KIR3DL1^-/~/+^), (B) CD69 expression of unstimulated and stimulated KIR3DL1-/~/+ Jurkat cells (C) 4.4-fold increase of MFI of CD69 (compared to unstimulated controls) on KIR3DL1ζ+ Jurkat cells coincubated with wildtype 721.221 (221) or cells transfected with HLA-B*08:01/HLA-B*57:01 (221-B57/221-B08) and treated without/with TUN (+T) (n = 10).

A Jurkat cell model stably expressing a chimeric receptor of the extracellular and transmembrane domain of KIR3DL1 fused to the cytoplasmic tail of CD3ζ, referred to as ‘KIR3DL1ζ‘, was employed to assess the functional consequences of glycosylation inhibition. KIR3DL1ζ^+^ ligand engagement by HLA-B*57:01 results in an activating signal that triggers CD69 expression. Jurkat cells were gated by size, granularity, CD3 expression and KIR3DL1ζ expression. Cells were classified as either KIR3DL1ζ^bright^, KIR3DL1ζ^dim^, KIR3DL1ζ^low^ (+/~/-) (Fig 3A). KIR3DL1ζ^-^ Jurkat cells were compared to untransduced Jurkat cells (data not shown) and no difference in activation after coincubation with HLA class I-expressing target cells was observed. Thus, KIR3DL1ζ^-^ Jurkats were used as a negative control (Fig 3B and 3C).

KIR3DL1ζ^+^ Jurkat reporter cells were potently stimulated by 221-HLA-B*57:01 cells. However, stimulation of KIR3DL1ζ^+^ Jurkat cells was abrogated by pre-treatment of 221-HLA-B*57:01 cells with TUN (4.4-fold CD69 MFI decrease, *p* = 0.003) (Fig 3C). As expected, 221-HLA-B*08:01 cells and untransduced 221 cells did not stimulate KIR3DL1ζ^+^ Jurkat cells, and treatment with TUN had no effect (Fig 3C). KIR3DL1ζ^–^(*i*.*e*. untransduced) Jurkat cells were not activated after-co-incubation with any of the tested target cells regardless of TUN treatment (Fig 3B and 3C). These results demonstrate that lack of *N*-glycosylation abrogates KIR3DL1:HLA-B*57:01 binding, and this has direct functional consequences on KIR3DL1ζ^+^ Jurkat cell function.

### Primary human KIR3DL1^+^ NK cells are ‘de-repressed’ upon encountering TUN-treated HLA-B*57:01^+^ target cells

**Fig 4:**
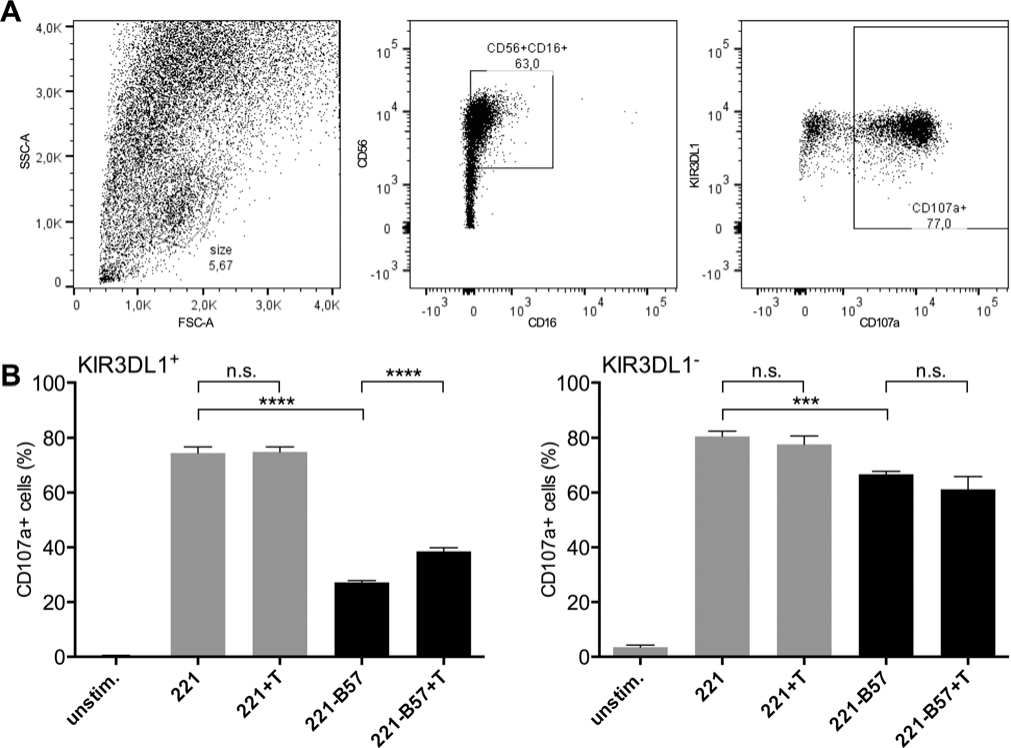
Disinhibition of KIR3DL1+ NK cell clones by TUN treatment (A) Gating strategy of NK cell clones by size, CD56, CD16, KIR3DL1 and CD107a expression (B) % of CD107a+ KIR3DL1+ and KIR3DL1- NK cell clones coincubated with wildtype 721.221 (221) or cells transfected with HLA-B*57:01 (221-B57/221-B08) and treated without/with TUN (+T) (n = 3).

We subsequently examined the effect of TUN on the interaction between primary human KIR3DL1^+^ NK cells and HLA-B*57:01^+^ target cells by assessing degranulation on KIR3DL1^+^ NK cells clones co-incubated with TUN-treated or untreated 221-HLA-B*57:01 cells. NK cell clones were gated for FSC, SSC and expression of CD16, CD56, KIR3DL1 and CD107a (Fig 4A). KIR3DL1^+^ NK cells exposed to 221 cells degranulated extensively (74.47% ± 2.20% CD107a+), but were significantly suppressed when exposed to 221-HLA-B*57:01 cells (27.20% ± 0.7% CD107a+, 2.7-fold decrease compared to the 221 samples, *p* < 0.0001), which was still significantly higher than unstimulated NK cells (0.46% ± 0.11% CD107a+) (Fig 4B). However, TUN pre-treatment of 221-HLA-B*57:01 cells resulted in a significant increase in degranulation (38.53% ± 1.37% CD107a+, 1.4-fold increase compared to 221-B57, *p* < 0.0001) compared to untreated 221-HLA-B*57:01 cells. KIR3DL1^-^ NK cells exposed to 221 cells (80.57% ± 1.87%) degranulated significantly more than when exposed to 221-B*57 cells (66.7% ± 1.05%, 1.2-fold decrease compared to 221, *p* = 0.0007). Co-incubation of target cells with TUN had no significant effect on KIR3DL1^-^ NK cells (221: *p* = 0.6874; 221-B57: *p* = 0.1629*)*. Thus, TUN treatment of 221-HLA-B*57:01 cells ‘de-repressed’ KIR3DL1^+^ NK cells that would normally have been suppressed by KIR3DL1 engagement of *N*-glycosylated HLA-B*57:01 proteins, suggesting a critical and functional role for HLA class I *N*-glycans in KIR binding and primary NK cell function modulation.

## Discussion

*N-*glycosylation is a critical step in post-translational protein modification with functional importance that is not yet fully understood. In the case of all HLA class I allotypes, a single, highly conserved *N-*glycosylation site is located at position N86 [14, 15], in close proximity to the highly polymorphic motif spanning from amino acids 77 to 83, which constitutes the Bw4 motif in HLA-Bw4 alleles and is critical for KIR3DL1 binding to these [25]. We therefore hypothesized that HLA class I *N-*glycosylation can modulate binding between HLA-Bw4 proteins and KIR3DL1. To test this hypothesis, we employed a panel of glycosylation enzyme inhibitors to modify or eliminate *N*-glycan structures in HLA-B*57:01-expressing cells and assessed HLA-B*57:01 expression and binding to KIR3DL1. We demonstrate that presence of the *N-*glycan on HLA-B*57:01 is critical for binding to KIR3DL1, and that inhibition of *N*-glycosylation in target cells can have functional consequences on KIR3DL1^+^ cell lines and primary KIR3DL1^+^ NK cells.

Inhibition of different glycosylation enzymes led to different effects in terms of HLA-B*57:01 expression and KIR3DL1 binding. The most striking effect was observed for TUN-induced inhibition of *N*-glycosylation, which increased cell surface expression of HLA-B*57:01 while abrogating KIR3DL1 binding. This was in contrast to all other inhibitors, which either increased (KIF, SWA), decreased (CSP, DNM), or did not significantly alter (AUS, STI) HLA-B*57:01 expression in a manner that correlated with increased, decreased, or unchanged KIR3DL1 binding, respectively. TUN-induced increase in HLA-B*57:01 expression was confirmed using two different antibodies—anti-HLA-Bw4 (clone: Bw4) and anti-pan-HLA class I (clone: W6/32)—demonstrating that the Bw4 motif, which is situated next to the *N*-glycosylation site, is still recognized, and that the HLA class I molecule is expressed as a complex bound to β_2_-microglobulin. We further demonstrated that loss of KIR3DL1-Fc binding to TUN-treated HLA-B*57:01^+^ target cells matched functional readouts using KIR3DL1ζ^+^ Jurkat reporter cells and primary human KIR3DL1^+^ NK cells. KIR3DL1ζ^+^ Jurkat cells were stimulated by co-incubation with 221-HLA:B*57:01 cells, yet this stimulation was almost completely abrogated by treating target cells with TUN prior to co-incubation. In line with this, primary KIR3DL1^+^ NK cells were potently inhibited by 221-HLA-B*57:01 cells, but were ‘de-repressed’ when 221-HLA-B*57:01 cells were pre-treated with TUN. These data indicates that *N*-glycosylation of HLA-B*57:01 plays a critical and functional role in KIR3DL1 binding and can modulate NK cell function, a finding that may extend to numerous other KIR:HLA interactions that have not yet been studied in the context of *N*-glycolysation.

**Fig 5:**
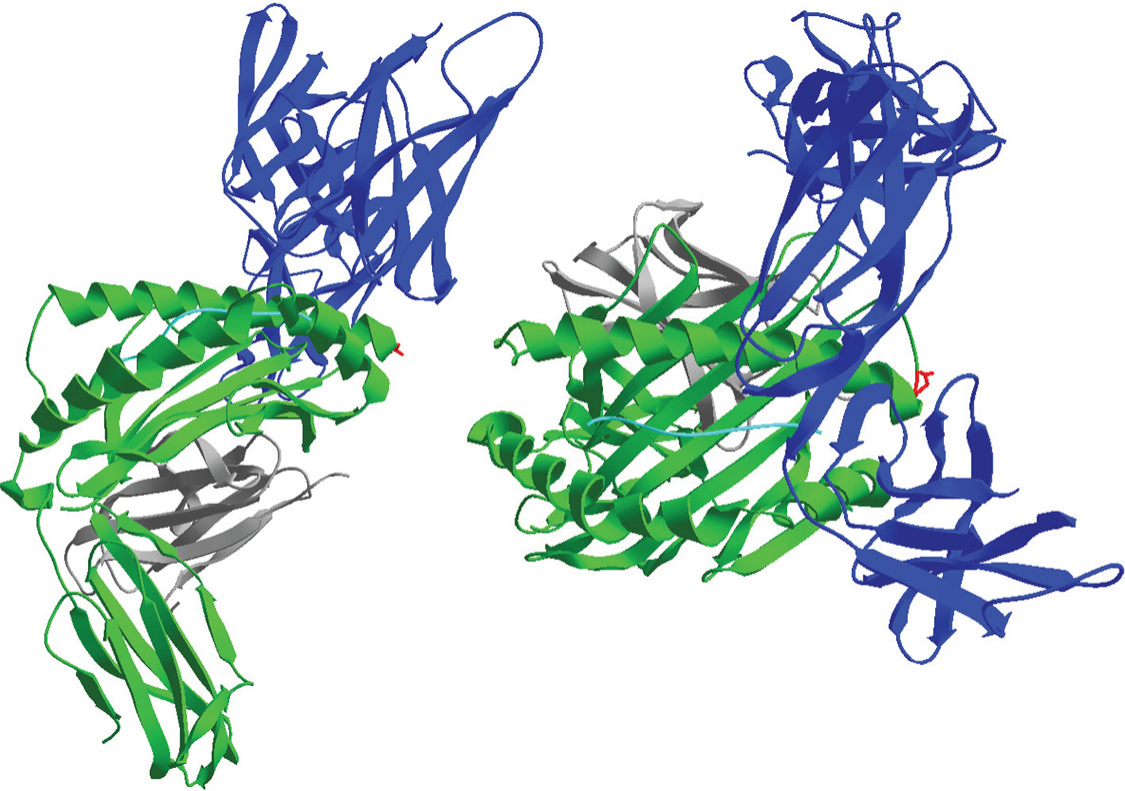
Secondary structure of HLA-B*57 and KIR3DL1: (Green) HLA-B*57, (Black) β2M, (Blue) KIR3DL1, (Cyan) Peptide bound in peptide-binding groove, (Red) Amino Acid N86, a site of N-glycosylation on HLA-B*57:01; Image generated using Swiss-PdbViewer 4.1.0 and a structure fie published by Vivien JP. [25, 41].

Reports of the importance of *N*-glycosylation for KIR:HLA binding are scarce, save for one study suggesting that the HLA class I *N*-glycan does not influence KIR binding [26]. In contrast, several studies reported the consequences of HLA class I *N*-glycosylation modifications on T-cell receptor (TCR) binding [23, 27–29]. Those reports all concluded that TCR binding to HLA class I (and MHC class I in mice) is independent of HLA class I glycosylation, given the relatively long distance between the TCR binding site and the HLA class I N86 glycan. KIR:HLA crystal structures, however, reveal a very close proximity between the KIR binding site on HLA class I and position N86, which in these structures is aglycosylated due to the production of these proteins in *E*. *coli* (Fig 5). This suggests that the HLA class I N86 glycan may be contacting KIR and influencing binding avidity. Of note, the one study that concluded that HLA class I glycosylation was not necessary for KIR binding was based on a generally assumed interaction between HLA-B*08:01 and an undiscovered inhibitory KIR, which later was found to not exist and only be the effects of the interaction between the inhibitory receptor NKG2A and HLA-E, which was not discovered at the time of the study [30]. Thus, to the best of our knowledge, our study is the first to implicate the HLA class I *N*-glycan as being critical for KIR:HLA binding, which may serve as another means of modulating the interaction between NK cell receptors and target cell ligands.

It has been demonstrated that the glycosylation pattern of several immune receptor-ligand pairs can be influenced in the setting of infection. In HIV-1 infection, a global shift in the glycosylation pattern of IgG has been observed, with HIV-1-specific antibodies displaying the most distinct glycosylation patterns {Ackerman, 2013 #357}[31, 32]. This shift in IgG glycosylation patterns can alter Fc receptor binding and is associated with improved antiviral activity and control of HIV-1, but has also been described for other viral and bacterial infections [31, 33]. Indeed, the *N*-glycan structure found on IgG is very similar to the HLA class I N86 glycan, and can be modified similarly by the addition of fucose, bisecting N-acetyl glucosamine, galactose, or sialic acid [34–36]. Furthermore, HIV-1 infection has been shown to alter glycosylation in host cells, and it is conceivable that HIV-1 might affect HLA class I glycosylation, either as a host response mechanism or a direct immunevasive tactic depending on whether HLA class I binding to NK cell receptors is enhanced or diminished by the altered glycosylation pattern. It has been suggested that other viruses have taken advantage of this level of regulation, as in the case of hepatitis C virus, which downregulates HLA class I expression in order to escape immune pressure, a process that is hypothesized to be due to altered glycosylation [37–40].

While much about the role of modified glycosylation patterns remains to be elucidated, our data demonstrates the importance of glycosylation in KIR:HLA binding and that removal of the glycan has a functional effect on the activation of NK cells. The extent to which pathogens and the immune system can exploit this mechanism to their advantage or whether this mechanism can be harnessed for therapeutic purposes means remains to be determined.

## Supporting Information

S1 Dataset Data for Fig 1 and S2 Fig: glycosylation enzyme inhibitor screening and titration. (XLSX)

S2 Dataset Data for Fig 2 and S1 Fig, anti-pan-HLA class I (W6/32), anti-Bw4 and KIR3DL1-Fc staining. (XLSX)

S3 Dataset Data for Fig 3, normalized MFI of CD69 of KIR3DL1ζ± Jurkat cells. (XLSX)

S4 Dataset Data for Fig 4, % of CD107a+ KIR3DL1± NK cell clones. (XLSX)

S1 Fig MFI of anti-pan-HLA class I (W6/32) staining of TUN and CSP treated 221 cells. (EPS)

S2 Fig (A) Effect of coincubating target cells with different concentrations of TUN on the CD69-expression of KIR3DL1ζ-Jurkat cells (n = 1), (B) Effect of coincubating target cells with different concentrations of CSP on the CD69-expression of KIR3DL1ζ-Jurkat cells (n = 1). (TIFF)
