# Supplementary figures and images for: Influence of Glycosylation Inhibition on the Binding of KIR3DL1 to HLA-B*57:01
Source: PLoS One. 2015 Dec 17;10(12):e0145324. doi: 10.1371/journal.pone.0145324 (PMC4683028; doi:10.1371/journal.pone.0145324)

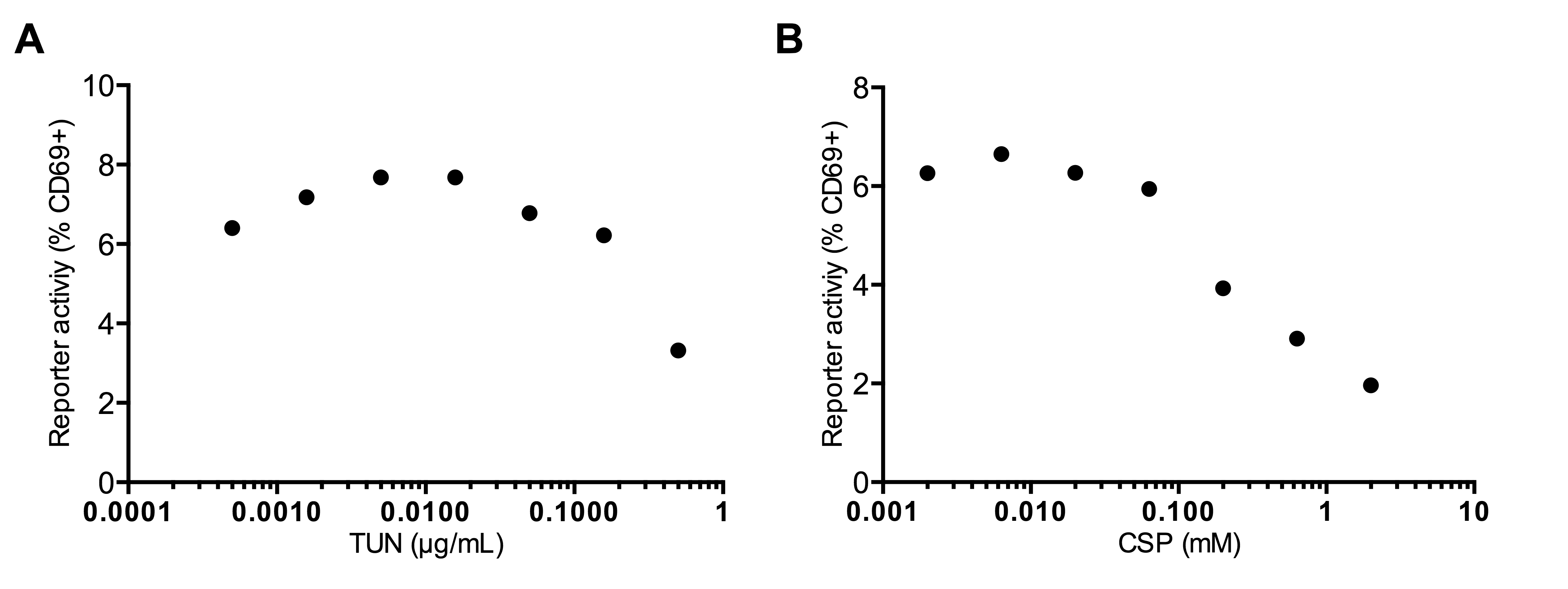

Supplement: S2 Fig — (TIFF) [file pone.0145324.s006.tiff]
